# Stereotactic Body Radiation Therapy after Chemotherapy for Unresectable Perihilar Cholangiocarcinoma: The STRONG Trial, a Phase I Safety and Feasibility Study

**DOI:** 10.3390/cancers13163991

**Published:** 2021-08-07

**Authors:** Rogier Baak, François E. J. A. Willemssen, Yvette van Norden, Ferry A. L. M. Eskens, Maaike T. W. Milder, Ben J. M. Heijmen, Bas Groot Koerkamp, Dave Sprengers, Lydi M. J. W. van Driel, Heinz-Josef Klümpen, Wilhelm den Toom, Merel S. Koedijk, Jan N. M. IJzermans, Alejandra Méndez Romero

**Affiliations:** 1 Department of Radiotherapy, Erasmus MC Cancer Institute, 3015 CN Rotterdam, The Netherlands; y.vannorden@erasmusmc.nl (Y.v.N.); m.milder@erasmusmc.nl (M.T.W.M.); b.heijmen@erasmusmc.nl (B.J.M.H.); w.dentoom@erasmusmc.nl (W.d.T.); a.mendezromero@erasmusmc.nl (A.M.R.); 2 Department of Radiology and Nuclear Medicine, Erasmus MC University Medical Center, 3015 CN Rotterdam, The Netherlands; f.willemssen@erasmusmc.nl; 3 Department of Medical Oncology, Erasmus MC Cancer Institute, 3015 CN Rotterdam, The Netherlands; f.eskens@erasmusmc.nl; 4 Department of Surgery, Erasmus MC University Medical Center, 3015 CN Rotterdam, The Netherlands; b.grootkoerkamp@erasmusmc.nl (B.G.K.); j.ijzermans@erasmusmc.nl (J.N.M.I.); 5 Department of Gastroenterology and Hepatology, Erasmus MC University Medical Center, 3015 CN Rotterdam, The Netherlands; d.sprengers@erasmusmc.nl (D.S.); l.m.j.w.vandriel@erasmusmc.nl (L.M.J.W.v.D.); 6 Department of Medical Oncology, Amsterdam University Medical Centers, University of Amsterdam, 1081 HV Amsterdam, The Netherlands; h.klumpen@amsterdamumc.nl; 7 Radiotherapeutisch Instituut Friesland, 8934 AD Leeuwarden, The Netherlands; M.S.Koedijk@skf-rif.nl

**Keywords:** perihilar cholangiocarcinoma, stereotactic body radiation therapy, chemotherapy

## Abstract

**Simple Summary:**

The role of radiotherapy in the treatment of perihilar cholangiocarcinoma has not yet been properly defined. In this prospective study, we therefore explored the addition to first-line chemotherapy of stereotactic body radiation therapy (SBRT) delivered in 15 fractions. Patients eligible for the study had been diagnosed with unresectable perihilar cholangiocarcinoma, and then had no progressive disease after completing treatment with 6–8 cycles of cisplatin-gemcitabine. Primary endpoints were feasibility and safety. Secondary endpoints were local control, progression-free survival, overall survival, and quality of life. As each patient completed the SBRT successfully and no dose-limiting toxicity was found, we consider this treatment to be both feasible and safe. The local control rate and overall survival were promising. However, due to the small sample size of this study, we urge the analysis of this treatment in a larger series of patients.

**Abstract:**

Background: In unresectable pCCA, the standard of care is palliative chemotherapy. We investigated the feasibility and safety of adding stereotactic body radiation therapy (SBRT) after chemotherapy. Methods: Patients with unresectable pCCA, stage T1-T4N0-N1M0, ECOG 0-1, having finished 6–8 cycles of cisplatin and gemcitabine without disease progression were eligible. SBRT was planned in 15 fractions of 3.0–4.5 Gy. The primary endpoints were feasibility (defined as completing SBRT as planned) and toxicity, evaluated within 3 months after SBRT (CTCAE v4.03). A conventional “3 + 3” design was used, corresponding to a sample size of 6 patients. Dose-limiting toxicity (DLT) was defined as grade ≥ 4 hepatobiliary or grade ≥ 3 gastrointestinal toxicity. The secondary endpoints, measured from the start of radiotherapy, were local control, progression-free survival, overall survival, and quality of life (QoL). ClinicalTrials.gov identifier: NCT03307538. Results: Six patients were enrolled between November 2017 and March 2020. SBRT was delivered as planned. All patients were treated with 60Gy (15 × 4.0Gy). No SBRT-related DLT was observed. The most common grade ≥ 3 toxicity was cholangitis (*n* = 5). The median follow-up was 14 months. The 12-month local control rate was 80%. We observed no substantial changes in QoL. Conclusion: In patients with unresectable pCCA with stable disease after palliative chemotherapy, adding SBRT is feasible and safe. The observed local control merits an additional evaluation of effectiveness.

## 1. Introduction

Cholangiocarcinoma (CCA) is the second most common primary liver tumor worldwide [1]. Approximately 50%–70% of all CCA arise at the hilar plate of the biliary tree; they are referred to as perihilar CCA [2]. The only potential curative treatment for patients with perihilar CCA is resection or liver transplantation. However, the large majority of patients present with unresectable disease at diagnosis [3,4,5].

The current standard of care for patients with unresectable and/or metastatic perihilar CCA is palliative chemotherapy consisting of 6–8 courses of cisplatin and gemcitabine in the first line and, recently, folinic acid, fluorouracil, and oxaliplatin in the second line [6,7,8].

The poor overall survival for patients with unresectable perihilar CCA treated with chemotherapy has led to studies of various local therapies, of which intraductal radiofrequency ablation, irreversible electroporation, and photodynamic therapy using temoporfin have been explored [9,10,11,12].

The role of radiotherapy in the treatment of perihilar CCA has not yet been properly defined, even though data from brachytherapy in combination with external beam radiotherapy (EBRT), as well as proton therapy, seem to show some activity [13,14]. 

To deliver high-radiation doses to patients with unresectable perihilar CCA, various groups have tried to achieve local control (LC) through the use of stereotactic body radiation therapy (SBRT). To date, however, most studies describe retrospective data in heterogeneous patient populations [15,16,17,18,19,20,21,22].

In the current study, we explored in a prospective way the feasibility and safety of SBRT delivered in 15 fractions to patients with unresectable perihilar CCA who had been treated with 6–8 cycles of cisplatin-gemcitabine and in whom no progressive disease was noted after the completion of this treatment. 

To the best of our knowledge, this is the first study to prospectively investigate this treatment combination for patients with perihilar CCA. 

## 2. Materials and Methods

### 2.1. Trial Design and Sample Size

This study was a single-center phase I feasibility and safety trial [23]. Approval was granted by the Medical Ethics Committee at Erasmus Medical Center (MEC-2017-386). The study was considered to be subject to the Medical Research Involving Human Subjects Act. The study was registered at ClinicalTrials.gov (identifier: NCT03307538) (accessed on 1 July 2020).

The study followed the “3 + 3” design, in which, after the first 3 patients had been included, the trial was temporarily put on hold for 3 months to observe the potential dose-limiting toxicity (DLT). DLT was defined as grade ≥ 4 hepatobiliary toxicity or grade ≥ 3 gastrointestinal toxicity occurring within a 3-month period after SBRT. If 2 or more of the initial 3 patients developed DLT, the trial was to be closed. If 0 or 1 of the initial 3 patients had DLT, another 3 patients could be included. If 0 or 1 of 6 patients developed DLT toxicity, it would be concluded that the current radiotherapy protocol was acceptable and could be considered for further research in this patient population.

### 2.2. Patient Population

To be eligible for this study, patients had to be diagnosed with perihilar CCA according to the criteria of the Mayo Clinic, Rochester, MN, USA [24]. The tumor had to be a single mass, and surgically unresectable. Patients had to be staged N0 or N1 (AJCC staging, seventh edition), and the latter was defined as lymph nodes that were radiologically suspect or pathologically confirmed. Patients had to be able to start SBRT within 12 weeks of completing 6–8 cycles of first-line chemotherapy (Cisplatin-Gemcitabine). Additional inclusion criteria were age ≥ 18 years; an ECOG performance status of 0 or 1; maximum liver cirrhosis of Child-Pugh grade A; bilirubin ≤ 1.5 times the upper limit of normal (ULN); AST/ALT ≤ 5 times ULN; platelets ≥ 50 × 10^9^/L; leucocytes > 1.5 × 10^9^/L; and hemoglobin > 6 mmol/L. Exclusion criteria were a prior surgery, tumor extension into the surrounding organ tissue, and distant metastasis or progression during or after chemotherapy. The material choice of the biliary stents was left to the discretion of the gastroenterologist. To be eligible for this study, patients were discussed by a multidisciplinary liver tumor board.

### 2.3. Study Objectives

The primary endpoints were (1) feasibility, defined as completing SBRT as planned and (2) toxicity as evaluated within 3 months of treatment according to the Common Toxicity Criteria for Adverse Events (CTCAE V4.03) [25].

Secondary endpoints were local control, progression-free survival, overall survival, and quality of life. Local control was defined as the time from the start of radiotherapy to local radiological progression (based on RECIST v1.1 [26]). Progression-free survival (PFS) was defined as the time from the start of radiotherapy to either local or distant radiological progression. Overall survival was defined as the time from the start of radiotherapy until a death from any cause. Quality of life (QoL) was assessed by means of (1) the EuroQol (EQ)-5D-5L (measure of health outcome in general population) and (2) the European Organization for the Research and Treatment of Cancer (EORTC) QLQ-C30 (QoL specific for patients with cancer), including the supplementary module EORTC QLQ-BIL21 (specific for CCA and gallbladder cancer). 

### 2.4. Radiation Treatment Preparation and Delivery

To deliver the highest possible dose to the tumor, we proposed a dose prescription using 15 fractions of 3.0–4.5 Gy, but not exceeding the widely accepted constraints and objectives in the organs at risk (OAR) (Table 1 and Table 2). This fractionation scheme had been tested in a multicenter retrospective study for intrahepatic CCA. We applied the same objective to the biliary tract, since the study reported limited biliary stenosis (9%). Within these constraints, the full planning target volume (PTV) was ideally irradiated with a dose of ≥60Gy (15 × 4.0 Gy). The intention was to deliver at least 60 Gy (biologically effective dose >80.5 Gy) to the largest possible portion of the PTV without violating hard OAR constraints [27]. No dose escalation was intended in this study.

Per patient, an experienced interventional radiologist implanted fiducials in the liver close to the tumor. The CT for radiotherapy planning was scheduled between 5 and 10 days after the intervention. No fiducials were implanted in the affected nodes of patients with lymph node involvement.

The gross target volume (GTV) was defined in a contrast-enhanced CT acquired in expiration and in a hepatic venous phase. Directly after this scan, and still in the venous phase, a CT was also performed in inspiration. A diagnostic gadolinium-enhanced liver MRI, with a standardized protocol, was acquired as part of the treatment preparation and was fused with the planning CT. The delineation was performed by an experienced radiation oncologist (AMR) and reviewed by an experienced hepatobiliary radiologist (FW). No additional margin was added around the GTV to generate the clinical target volume for both tumor and lymph nodes. The organs at risk (OAR) were delineated in the planning CT acquired in expiration. Also, limiting OAR located very close to the GTV (i.e., stomach, duodenum, bowel, gallbladder) or in the GTV (i.e., central biliary tree) were delineated in the inspiration CT. Both contours were considered for planning. 

To generate the PTV, the information acquired from a 4DCT scan (fiducial markers) and from the inspiration/expiration CT (tumor, stents) was used to establish the margin around the GTV (tumor and lymph nodes if needed). This margin aimed at ensuring that, despite geometrical uncertainties (such as respiratory tumor motion, inter-fraction, intra-fraction, and set-up uncertainties), the fullest extent of the GTV would be irradiated with a sufficient dose. Planning OAR constraints and objectives for PTV and OAR are shown in Table 1 and Table 2. The treatment was delivered with a CyberKnife using a tumor-tracking technique (Synchrony-CyberKnife, Accuray, Sunnyvale, CA, USA).

### 2.5. Follow-Up

Follow-up visits were scheduled at 1, 3, 6, 9, 12, 18, and 24 months after treatment until disease progression. At each visit, the toxicity and performance status were scored and a CT scan was acquired. Moreover, bilirubin, alanine aminotransferase (ALT), aspartate aminotransferase (AST), and gamma-glutamyltransferase (GGT) blood levels were measured. If distant disease progression occurred first, we recalled imaging examinations to determine whether local recurrence had occurred during follow-up. The overall survival was assessed with information collected through general practitioners or referral hospitals. Patients were asked to fill out QoL questionnaires (EORTC QLQ-C30, EQ-5D-5L, and EORTC QLQ-BIL21) at 1, 3, 6, 9, 12, and 24 months. QoL was assessed until disease progression [23]. After disease progression, further treatment was left to the discretion of the referring specialist.

### 2.6. Statistical Analysis

The Kaplan–Meier estimate was used to assess LC, PFS, and OS. The date of the last known radiology examination was regarded as the last follow-up date for LC. The calculations were conducted using STATA, v15.

## 3. Results

### 3.1. Patient Population

**Figure 1:**
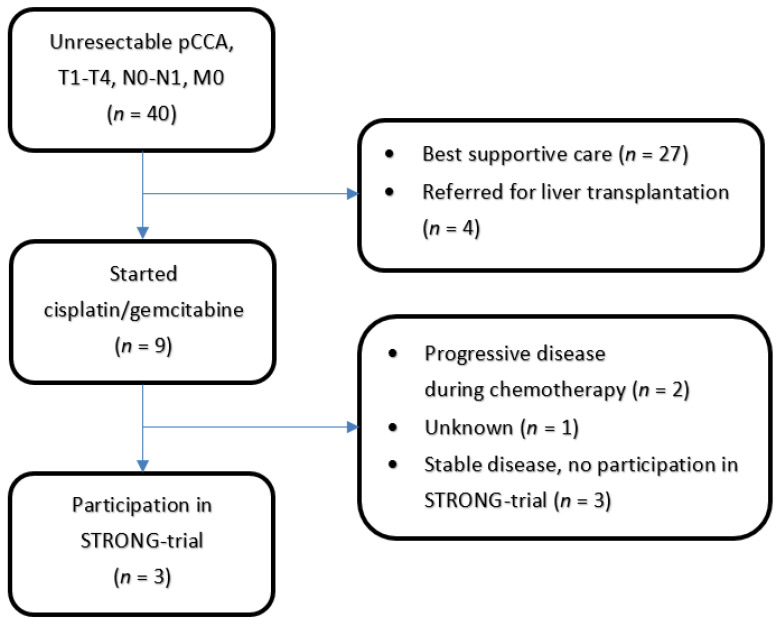
Patients with unresecectable pCCA referred from Erasmus Medical Center during the inclusion period for the STRONG-trial. Of the 6 patients included in the trial, 3 patients were referred from another hospital after chemotherapy and were not included in this overview.

Six patients were enrolled between 1 November 2017 and 30 March 2020. The last scheduled follow-up visit took place on 30 June 2020. Three patients were treated with chemotherapy at the Erasmus Medical Center, and the other three patients were referred from another center after chemotherapy. The patient selection is shown in Figure 1. All six patients underwent chemotherapy as planned in the form of cisplatin and gemcitabine (6–8 courses). Patient characteristics are presented in Table 3.

Each patient was staged pre-chemotherapy with T4 perihilar CCA. All tumors were considered unresectable due to vascular involvement. N-staging varied between N0 (*N* = 3) and N1 (*N* = 3); all patients were staged M0. The staging did not change after chemotherapy. The Bismuth classification varied between II (*N* = 2), IIIA (*N* = 1), IIIB (*N* = 1), and IV (*N* = 2). The median tumor size was 26 mm in the axial plane on diagnostic imaging. Each patient had an ECOG performance status of 1. Five patients (83%) had pathologically confirmed perihilar CCA from brushing. The patient without pathology confirmation had qualified for the diagnosis of perihilar CCA on the basis of the criteria of the Mayo Clinic [24]. IgG levels were within normal values.

At the start of radiotherapy, patients either had a metal hepatobiliary stent (*N* = 3), a plastic hepatobiliary stent (*N* = 2), or no hepatobiliary stent (*N* = 1) in situ. No patients had liver cirrhosis. Baseline adverse events are shown in Table 4.

### 3.2. Radiation Treatment

Radiotherapy was started after a median of 66.5 days (range: 42–78 days) after the end of chemotherapy. The treatment was delivered in 15 daily fractions with an overall treatment time ranging from 20 to 22 days (as seen in Table 5). All patients completed the treatment as planned. Patients received no further treatment after SBRT completion until progression.

A total dose of 60Gy was delivered at the 80% isodose, and the maximum point dose was 75Gy. No constraints were violated, and all objectives were respected. As sparing of the OARs was prioritized, a coverage ≥ 95% PTV with 60Gy could not be achieved. The median coverage of the PTV at 60Gy was 82.4% (range 75.7%–89.6%), and the median coverage of PTV at 45Gy was 95.1% (range 89.1%–99.5%).

### 3.3. Safety/Toxicity

No SBRT-related DLT was observed. The maximum grade toxicities during follow-up are shown in Table 6. Hepatobiliary toxicity grade 3 (cholangitis leading to unplanned hospitalization for non-surgical intervention) was found eight times. These adverse events were distributed over five patients, and ranged from one to four events per patient. Each episode of cholangitis could be reversed by improving the biliary drainage. The one patient who did not suffer from cholangitis after treatment had not required any endoscopic procedure for biliary drainage prior to treatment. Other observed grade ≥ 3 toxicities (investigations) were elevated ALT (*N* = 2 with grade 3) and elevated bilirubin (*N* = 1 with grade 3). The two patients with grade 4 GGT elevation (33%) had grade 3 GGT elevation at baseline. We attributed the grade 4 GGT elevation to cholangitis and progressive disease (liver metastasis) in one patient. The other patient was asymptomatic and had a history of alcohol abuse.

### 3.4. Efficacy Endpoints

**Figure 2:**
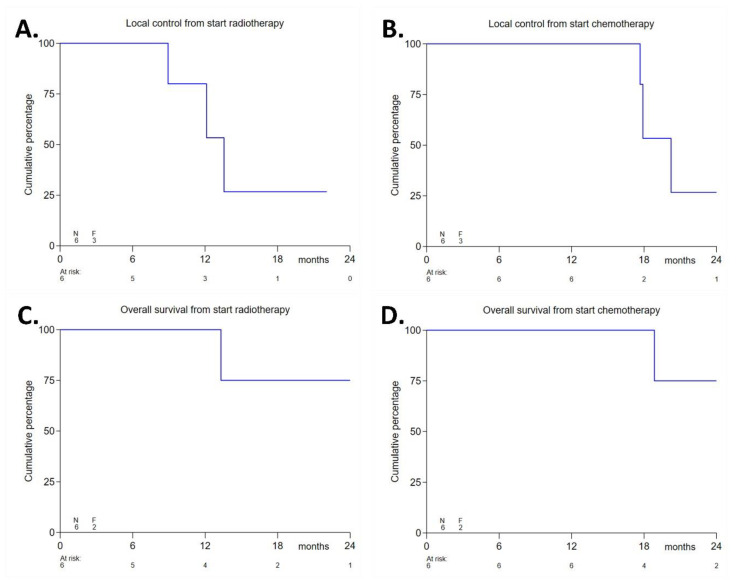
Efficacy endpoints: Local control from the start of radiotherapy (**A**), local control from the start of chemotherapy (**B**), overall survival from the start of radiotherapy (**C**), and overall survival from the start of chemotherapy (**D**).

The from the start of radiotherapy was 14 months (range 6–25 months), and the median follow-up from the start of chemotherapy was 21 months. The median local control was 14 months from the start of radiotherapy (for the respective Kaplan–Meier plots, see Figure 2A,B) and 20 months from the start of chemotherapy. The 1-year local control rate from the start of radiotherapy was 80%. The median progression-free survival was 14 months from the start of radiotherapy and 18 months from the start of chemotherapy. Distant disease progression was observed as peritoneal metastases/ascites (*N* = 3) and/or liver metastases (*N* = 1). Three patients with distant metastases had positive lymph-nodes at baseline. The course of these events is shown in Table 7. After a median follow-up of 14 months, four patients (67%) were still alive, whereas two patients (33%) had died due to progressive disease after 13.3 and 24.9 months from the start of radiotherapy, respectively. The median overall survival was not reached. The 1-year overall survival rate from the start of radiotherapy was 100%. Figure 2C,D shows the respective Kaplan–Meier plots of overall survival from the start of chemotherapy and from the start of radiotherapy.

### 3.5. Quality of Life

**Figure 3:**
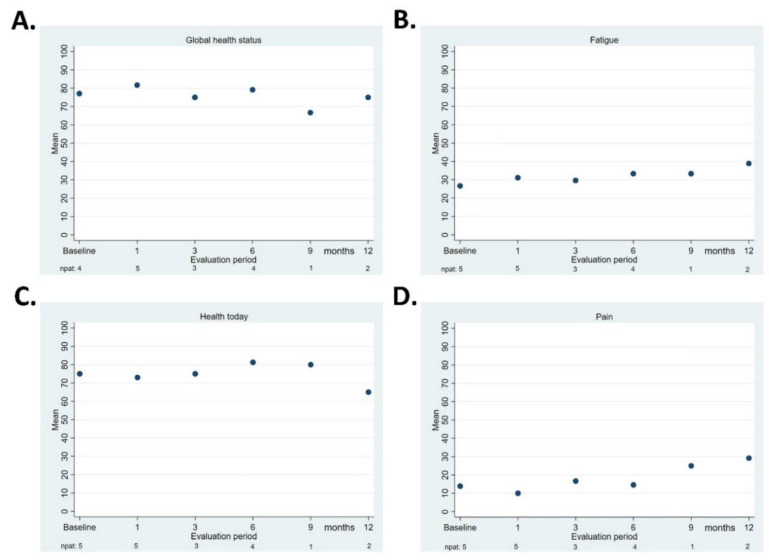
Quality of life, median score at each evaluation. QLQ-C30 “global health status” (**A**), EORTC QLQ-C30 “fatigue” (**B**), EuroQol-5D-5L “health today” score (**C**), and QLQ-BIL21 “pain” (**D**). The number of patients who responded is shown underneath each follow-up point.

Figure 3A–D presents the QLQ-C30 “global health”, QLQ-C30 “fatigue”, EQ-5D-5L “health today”, and QLQ-BIL21 “pain” scores. Due to the small number of patients, no statistical comparisons were performed between the baseline and the various evaluation points.

## 4. Discussion

Our trial of SBRT after first-line cisplatin and gemcitabine-containing chemotherapy in patients with unresectable perihilar CCA was designed to assess the feasibility and safety of this novel approach. The feasibility of SBRT was shown by the fact that all patients completed the SBRT as planned, and its safety by the absence of SBRT-related dose-limiting toxicity.

Due to the presence of OAR—such as the duodenum or stomach close to the GTV, or the presence of a dose-limiting structure such as the central biliary tract within the GTV—many difficulties are involved in delivering high RT doses in the perihilar region. As patient safety was our main priority, we attached greater importance to respecting the OAR constraints and objectives than to achieving a higher PTV coverage.

Although we observed no SBRT-related dose-limiting toxicity, five patients had a total of eight episodes (1–4 per patient) of cholangitis (grade 3 hepatobiliary toxicity) after treatment. These episodes could be resolved with improved biliary drainage. All five of these patients had undergone stent placement before SBRT (see Table 6). The 83% incidence of cholangitis is in striking contrast with the 0–38% incidence rate reported in other SBRT studies in patients with perihilar CCA [16,17,19]. The OAR objective we used for the central biliary tract was taken from a study of SBRT delivered in 15 fractions for intrahepatic CCA, which reported 4% cholangitis and 9% biliary stenosis [27]. When reviewing the dose distributions we had planned, we found that this objective had been respected in every patient. However, compared to intrahepatic CCA, perihilar CCA affects the central biliary tract and causes more significant obstruction and cholangitis. 

The three SBRT studies on perihilar CCA reported no objective or constraint for the central biliary tract. Kopek et al. observed no cholangitis in 27 patients, even though the dose in the central biliary tract had most probably exceeded the constraint applied in our study [16]. Although Polistina et al. observed no cholangitis in 10 patients during treatment, they did not report any results on cholangitis after treatment [17]. The dose delivered in the central biliary tract was probably comparable to that in our study. Momm et al. reported 38% cholangitis in 13 patients, in whom the dose delivered in the central biliary tract was probably lower than ours [19]. The retrospective nature of two of these studies may also have made it difficult to assess toxicity. 

In a randomized controlled trial in which 174 extrahepatic CCA patients were treated with stenting alone or with stenting and RFA, Gao et al. reported cholangitis during a median follow-up of 31.8 months in 37.9% of patients in the RFA-group and 36.8% of patients in the non RFA-group [9]. As the exact contribution of radiotherapy to the occurrence of hepatobiliary toxicity thus remains uncertain, it is a point of interest for further research. 

While we observed no grade ≥ 3 acute or late gastro-intestinal toxicity, including duodenal ulcers and stenosis, the range reported in the SBRT studies published on perihilar CCA was 0–26%, [16,17,19]. The absence of this toxicity in our study may have resulted from our strict adherence to the OAR constraints.

With regard to the secondary endpoint of antitumor activity, the 1-year local control rate in our group of patients was 80%, a figure that seems to compare well with the 78–84% in the other SBRT studies in perihilar CCA [16,17,19]. The median progression-free survival from the start of radiotherapy was 14 months. However, our patient selection may have been slightly biased: all patients had extensive vascular involvement, which is considered prognostically poor [33], and thus made them unsuitable for another ongoing study with electroporation therapy [10]. During follow-up, distant metastases developed in all patients in our population who had been diagnosed with lymph node metastases at baseline (see Table 6). Although our sample size was too small to allow for direct conclusions, further research should take this observation into account: it may help to select patients who would not benefit from the addition of a local treatment modality to standard first-line chemotherapy.

In general, patients with unresectable perihilar CCA have a poor prognosis, chemotherapy currently being the only option to increase overall survival, and then only to a limited extent: the overall survival is only 12.8 months, even for those who are eligible to receive cisplatin and gemcitabine [8]. The median overall survival data in studies focusing on the application of any form of radiotherapy in patients with locally advanced unresectable perihilar CCA ranged from 10.6 to 35.5 months [16,17,19]. Five of the studies with all forms of CCA reported an OS ≥ of 15 months, and three of them reported an OS > of 24 months, with a total range of 10–35.5 months. The 1-year OS rate ranged from 45% to 87%. Although our 1-year OS rate was 100%, our results cannot yet be compared with the median OS these studies reported [15,16,17,18,19,20,21,22].

Other radiotherapy techniques have been used to treat patients with unresectable CCA. Autorino et al. reported a 1-year LC rate of 82% and a median OS of 16 months in patients with distal and perihilar CCA treated with EBRT, concurrent chemotherapy, and brachytherapy [13]. Makita et al. treated 28 CCA patients (six perihilar CCA) with proton therapy alone, and reported a 1-year LC rate of 67.7%, and a median OS of 12 months [14]. Again, these data seem to be comparable to those observed in our study.

The QoL assessments in our study were the most reliable in the first 12 months; later, due to disease progression and the completion of the study, the number of respondents decreased. Even though the small number of patients makes it difficult to draw any solid conclusions, there were no signs of negative impact on scores for “global health,” “health today,” “fatigue,” and “pain.”

## 5. Conclusions

Our study on the feasibility and safety of SBRT after chemotherapy in patients with unresectable perihilar CCA is the first prospective study on this topic. Its prospective nature allowed us to deliver a consistent SBRT regimen and to determine all outcomes reliably. However, because its small sample size is a serious limitation, it is essential that this treatment is further analyzed in a larger series of patients.

## Figures and Tables

**Table 1:** Organs at risk constraints.

Organs at Risk	Hard Constraints
Healthy liver	≥700 mL liver-GTV,
	dose <25.5 Gy [28]
	If cirrhosis is present: NTCP
	liver-GTV ≤ 5% [29] and >800 mL
	liver-GTV, dose <31.5 Gy [30]
Stomach Duodenum Small and large bowel (combined in one structure when needed)	Max point dose <57 Gy [31] Volume receiving ≥41 Gy should be ≤5 cc
Esophagus	Max point dose ≤50.25 Gy [32]
Spinal cord	Max point dose ≤33.8 Gy [28]
Kidney	2/3 right kidney <25.5 Gy [31]

GTV: Gross Tumor Volume, NTCP: Normal Tissue Complication Probability.

**Table 2:** Organs at risk objectives.

Organs at Risk	Objectives
Central biliary tract	Less than 0.5 cc ≥ 70 Gy
	(NRG-GI001—http://www.cancer.gov/clinicaltrials) (accessed on 1 July 2020)
Heart	Max dose < 57 Gy
	(RTOG 1112—http://www.cancer.gov/clinicaltrials) (accessed on 1 July 2020)
Gallbladder	Max dose < 86.7 Gy
	(RTOG 1112—http://www.cancer.gov/clinicaltrials) * (accessed on 1 July 2020)
Skin (external contour)	Less than 0.5 cc ≥ 50.25 Gy
	(RTOG 1112—http://www.cancer.gov/clinicaltrials) (accessed on 1 July 2020)

* In practice, a max dose of ≤ 63 Gy was used.

**Table 3:** Baseline Characteristics.

Age in Years	
Median	65
Range	37–73
**ECOG Performance Status**	
1	6 (100%)
**Sex**	
Male	3 (50%)
Female	3 (50%)
**Previous Chemotherapy for Cholangiocarcinoma?**	
Gemcitabine + Cisplatinum	6 (100%)
**Number of Cycles Administered**	
6	2 (33%)
8	4 (67%)
**Response to Previous Chemotherapy (RECIST v1.1)**	
Partial response	1 (17%)
Stable disease	5 (83%)
**Stent in Situ at Start of Radiotherapy**	
Metal	3 (50%)
Plastic	2 (33%)
No	1 (17%)
**cTNM-Classification before and after Chemotherapy**	
T4	6 (100%)
**cTNM-Classification before and after Chemotherapy**	
N0	3 (50%)
N1	3 (50%)
**Bismuth–Corlette Classification**	
II	2 (33%)
IIIa	1 (17%)
IIIb	1 (17%)
IV	2 (33%)
**Tumor Size in Millimeters**	
Median	26
Range	21–36
**Pathologic Diagnosis of Tumor**	
Adenocarcinoma	5 (83%)
Unknown	1 (17%)

**Table 4:** Maximum grade toxicity (CTCAE v4.03) at baseline.

Fatigue	
0	1 (17%)
1	5 (83%)
**Pain**	
0	5 (83%)
1	1 (17%)
**Infection**	
0	5 (83%)
2	1 (17%)
**ALT Elevation**	
0	4 (67%)
1	2 (33%)
**AST Elevation**	
0	3 (50%)
1	3 (50%)
**Gamma-GT Elevation**	
0	2 (33%)
1	1 (17%)
3	3 (50%)
**Bilirubin Elevation**	
0	6 (100%)

ALT: Alanine aminotransferase, AST: Aspartate aminotransferase, Gamma-GT; Gamma-glutamyltransferase.

**Table 5:** Treatment characteristics.

Time from end of chemotherapy to start of radiotherapy in days	
Median	67
Range	42–78
**Overall radiotherapy treatment time in days**	
Median	21.5
Range	20–22
**GTV Coverage with 60Gy (%)**	
Median	91.7
Range	86.8–98.6
**GTV Coverage with 45Gy (%)**	
Median	99.4
Range	99.3–100
**GTV D2 (Gy)**	
Median	73.1
Range	70.5–73.4
**GTV D50 (Gy)**	
Median	67.1
Range	65.3–69.4
**PTV Coverage with 60Gy (%)**	
Median	82.4
Range	75.7–89.6
**PTV Coverage with 45Gy (%)**	
Median	95.1
Range	89.1–97.6

GTV: Gross tumor volume, PTV: Planning target volume, D2 and D50 are minimum dose to 2% and 50% volumes, respectively.

**Table 6:** Maximum grade toxicity (CTCAE v4.03) during follow-up.

Hepatobiliary Disorders	
0	1 (17%)
3	5 (83%)
**Fatigue**	
0	2 (33%)
1	4 (67%)
**Nausea**	
0	3 (50%)
1	3 (50%)
**Vomiting**	
0	5 (83%)
1	1 (17%)
**Diarrhea**	
0	5 (83%)
1	1 (17%)
**Pain**	
0	1 (17%)
1	4 (67%)
2	1 (17%)
**ALT elevation**	
0	1 (17%)
1	2 (33%)
2	1 (17%)
3	2 (33%)
**AST elevation**	
1	4 (67%)
2	2 (33%)
**Gamma-GT elevation**	
1	1 (17%)
2	1 (17%)
3	2 (33%)
4	2 (33%)
**Bilirubin elevation**	
0	4 (67%)
1	1 (17%)
3	1 (17%)

ALT: Alanine aminotransferase, AST: Aspartate aminotransferase, Gamma-GT: Gamma-glutamyltransferase.

**Table 7:** Patient overview.

PID	TNM- Staging	Bismuth–Corlette Staging	PTV Coverage 60Gy (%)	Type of Stent (Time Between Last Placement and Start of RT in Months)	Number of Cholangitis Episodes during FU (Time from Start of RT in Months)	Local Recurrence during FU (in Months)	Distant Metastasis during FU (in Months)	Death during FU (in Months)
1	cT4N0M0	II	80.1	Metal (4.1 m)	2 (10.8 m, 12.1 m)	X (13.6 m)	X (20.8 m)	X (24.9 m)
2	cT4N0M0	IIIB	81.0	Metal * (1.5 m)	4 (1.1 m, 12.4 m, 15.2 m, 17.3 m)	-	-	-
3	cT4N1M0	IV	75.7	Plastic (7.5 m)	2 (1.3 m, 9.7 m)	X (12.1 m)	X (3.8 m)	X (13.3 m)
4	cT4N1M0	IIIA	87.5	Plastic (7.5 m)	1 (4.3 m)	X (8.9 m)	X (12.3 m)	-
5	cT4N0M0	IV	89.6	-	-	-	-	-
6	cT4N1M0	II	83.7	Metal (11.4 m)	1 (1.9 m)	-	X (1.7 m)	-

* With a metal stent in place, a plastic stent was added. PTV: Planning target volume.

## Data Availability

Research data are stored in an institutional repository. Data are only available on request and to the last author due to privacy restrictions.
